# TGFβ1 induces bone formation from BMP9-activated Bone Mesenchymal Stem Cells, with possible involvement of non-canonical pathways

**DOI:** 10.7150/ijms.45786

**Published:** 2020-07-02

**Authors:** Huan Yao, Yulong Zou, Ke Yang, Liangjun Yin, Yang Liu, Ruidong Li

**Affiliations:** 1 The First Affiliated Hospital, Chongqing Medical University, Chongqing, China.; 2 Department of Orthopaedic Surgery, the Second Affiliated Hospital, Chongqing Medical University, Chongqing, China.; 3 The Children's Hospital, Chongqing Medical University, Chongqing, China.

**Keywords:** Osteogenesis, TGFβ1, BMP9, HSP47, Bone mesenchymal stem cell

## Abstract

Reconstruction of bone defects is one of the most substantial and difficult clinical challenges in orthopedics. Transforming growth factor beta 1 (TGFβ1) might play an important role in stimulating osteogenic differentiation of bone morphogenetic protein 9 (BMP9)-induced C3H10T1/2 mesenchymal stem cells. In our current study, we examined the potential synergy between TGFβ1 and BMP9 in promoting the osteogenesis of C3H10T1/2 cells, and whether such effects could contribute to bone formation *in vivo*. Our experiment data indicated that TGFβ1 could increase the expression of osteogenic markers and the formation of mineralized calcium nodules in, while suppressing the proliferation of, BMP9-induced C3H10T1/2 cells. Furthermore, mice intramuscularly injected with BMP9/TGFβ1-transduced C3H10T1/2 cells into the gastrocnemius muscle on their tibiae developed ectopic bone masses with more mature osteoid structures, compared to those grafted with cells expressing BMP9/RFP. Subsequent mechanistic studies found that TGFβ1-induced enhancement of osteogenesis in BMP9-overexpressing C3H10T1/2 cells was accompanied by augmented expression of heat shock protein 47 (HSP47), a collagen-specific molecular chaperone essential for collagen biosynthesis, and can be attenuated by pirfenidone, a known anti-fibrotic inhibitor. Interestingly, protein microarray analysis suggested that TGFβ1/BMP9-dependent osteogenesis of C3H10T1/2 cells seemed to involve several non-canonical signaling pathways such as Janus kinase-signal transducer and activator of transcription, phosphoinositide-3-kinase-protein kinase B, and mitogen-activated protein kinase. These results provided further evidence that TGFβ1 could promote bone formation from BMP9-induced C3H10T1/2 cells and shed important light on the underlying molecular mechanisms.

## Introduction

Reconstruction of bone defects caused by trauma is one of the most substantial and difficult clinical challenges in orthopedics 1, 2. Recently, bone tissue engineering, which uses autologous or allogeneic tissues for bone defect repair, has emerged as a promising alternative to grafting 3. There is consensus that success in bone tissue engineering entails an optimal combination of stem cells, growth factors and scaffolds. Bone marrow-derived mesenchymal stem cells (BMSC) have been widely viewed as promising candidates for bone tissue engineering because of their excellent pluripotency, affinity to plastic surface, and the ease with which they can be harvested and cultured 4, 5. Extensive research has lent support to the potential efficacy of BMSCs for restoring various bone defects in laboratory animals such as mice 6, 7 and rats 8, 9. The C3H10T1/2 cell line, which is isolated from C3H mice and immortalized, is one of the most representative acknowledged BMSCs at present 10, 11. Although clinical evaluation of BMSCs is still exploratory, Gan et al. has demonstrated that they can effectively promote posterior spinal fusion when combined with porous β-tricalcium phosphate 12.

Bone morphogenetic protein 9 (BMP9) is a relatively obscure member of the BMP family and has recently been found to be a key regulator of osteogenic stem cell differentiation 13, 14. In fact, there is evidence that BMP9 could be one of the most osteogenic BMPs both *in vitro* and *in vivo* 15. For example, several studies have indicated that up-regulation of BMP9 could dramatically increase the activity of alkaline phosphatase (ALP) and promote the deposition of calcium in C3H10T1/2 cells 9-11 and C2C12 cells 16. Similar effects have also been observed in animal models 17. Although the mechanisms responsible for the osteo-inductive activities of BMP9 have not been fully elucidated, recent investigations suggested that the signaling pathway involves receptor binding of BMPs 18, 19 followed by the phosphorylative activation of mothers against decapentaplegic homolog (Smad), which are well-established osteogenic modulators 20.

Transforming growth factor beta 1 (TGFβ1), a master regulator of cell survival, proliferation and differentiation, has also been shown to play an important role in stimulating the growth of bone 21. Unlike BMP9, while TGFβ1 alone has been shown to induce the osteogenic differentiation of oriented osteogenic ancestral cells, there is, to the best of our knowledge, no evidence that it can also exert the same effects on BMSCs 22, 23. TGFβ1 and BMP9 signaling pathways share a striking resemblance in that both are initiated by ligand binding to a heterodimeric complex of transmembrane receptor serine-threonine kinases 19, prompting speculation of possible cross-talk between them. Using supernatant obtained from a culture of TGFβ1-overexpressing HCT116 cells, we have previously discovered that TGFβ1 could exert an osteo-stimulatory effect on BMP9-transduced C3H10T1/2 cells at low concentrations, but would inhibit osteogenesis at high concentrations 22. These findings thus suggested that much remains to be learned about the interaction between the TGFβ1 and the BMP9 signaling pathways in BMSCs, particularly in an *in vivo* setting. In the current study, we demonstrated that the same osteo-inductive effect could be observed in BMP9-stimulated C3H10T1/2 cells upon adenoviral transduction of TGFβ1 gene. Engraftment of these C3H10T1/2 cells into the gastrocnemius muscle on the tibiae of mice could indeed lead to augmented bone formation and maturation. Heat shock protein 47 (HSP47) was found to be up-regulated and could play a key role in promoting the development of osteoid structures by activating its downstream target collagen type 1 (COL1). Interestingly, Western blotting and protein microarray results revealed the activation of several non-Smad signaling pathways, including Janus kinase (JAK)-signal transducer and activator of transcription (STAT), phosphoinositide-3-kinase (PI3K)-protein kinase B (Akt), and mitogen-activated protein kinase (MAPK). Taken together, the results offered useful insights into the molecular mechanisms responsible for TGFβ1-dependent osteo-induction of BMSCs.

## Methods

### Reagents and cell culture

HEK293 and C3H10T1/2 cell lines (ATCC, Manassas, Virginia, USA) were maintained in complete Dulbecco's Modified Eagle's Medium (DMEM) and complete Basal Medium Eagle (BME) (Gibco, Thermo Fisher Scientific, Waltham, Massachusetts, USA), respectively, at 37 °C under a humidified atmosphere containing 5% CO_2_.

Pirfenidone was purchased from AbMole Bioscience, USA, and dissolved in DMSO. For inhibition of HSP47, the above stock solution was added to suspensions of C3H10T1/2 cells to a final concentration of 1000 µg/mL as previously described 24.

### Construction of recombinant adenoviruses

Recombinant adenoviruses were generated using the AdEasy Adenoviral Vector System 25. Briefly, the coding sequences of human TGFβ1 and mouse BMP9 were inserted into a red fluorescent protein (RFP)-labeled adenoviral shuttle vector and a green fluorescent protein (GFP)-labeled adenoviral shuttle vector, respectively. The resultant constructs were linearized and transfected into HEK293 cells by Lipofectamine 2000 (Thermo Fisher Scientific, Waltham, Massachusetts, USA) to produce adenoviruses expressing both TGFβ1 and RFP (AdTGFβ1-RFP) or both BMP9 and GFP (AdBMP9-GFP). RFP- (AdRFP) and GFP-only (AdGFP) control viruses were constructed by transfecting HEK293 cells with the corresponding insert-free vectors.

### Optimization of C3H10T1/2 cell infection with AdTGFβ1-RFP

C3H10T1/2 cells were grown to the exponential stage, seeded to a 25 cm^2^ culture flask, and then infected with AdBMP9-GFP for 10 h. The infection efficiency of AdBMP9-GFP was set to 20% as it has been previously found that excessive up-regulation of BMP9 could mask the osteogenic effects of TGFβ1 14, 22. Then, the cells were seeded to 24-well plates and infected with different titers of AdTGFβ1-RFP. Transduction efficiency of TGFβ1 was estimated by measuring the percentage of RFP-positive C3H10T1/2 cells under a fluorescence microscope. The cells were cultivated in serum-free BME at 37 °C under 5% CO_2_ for 14 d. The concentration of TGFβ1 in the culture, which correlated to transduction efficiency, was measured by using a TGFβ1 ELISA Kit (Enzo Life Sciences, Farmingdale, New York, USA) at the indicated days. A standard curve was generated by serially diluting a starting TGFβ1 solution from 4000ng/mL to 62.5ng/mL. The obtained standard formula was: concentration (ng/mL) = [409.91 * OD^2^ + 741.75 * OD + 2.0791] / 500.

### Quantitation of ALP activity

C3H10T1/2 cells were consecutively infected with AdBMP9-GFP and AdTGFβ1-RFP (or AdRFP in the control group) in 24-wells plates as described above, with a 10-h interval in between. At the indicated days, the cells were harvested, stained using an Alkaline Phosphatase Kit (Sigma-Aldrich, St. Louis, Missouri, USA) following the manufacturer's instructions, and observed under a bright-field microscope 25.

### Alizarin red staining

Infected C3H10T1/2 cells were treated as described above and grown at 37 °C for 17 d in serum-free BME supplemented with 10 mM β-glycerophosphate and 50 μg/mL ascorbic acid. At the indicated days, the cells were harvested, fixed with 0.05% (v/v) glutaraldehyde at room temperature for 10 min, rinsed with distilled water, and incubated with 0.4% Alizarin Red S (Solarbio, Beijing, China) for 5 min. After removing the excess dye by rigorous washing with distilled water, the stained cells were visualized under a bright-field microscope to analyze the formation of mineralized calcium nodules.

### Western blotting

Briefly, cells were lysed in Laemmli buffer consisting of 60 mM Tris-HCl buffered at 6.8, 2% (w/v) sodium dodecyl sulfate (SDS), 10% (w/v) glycerol and 0.01% (w/v) bromophenol blue. The resultant lysate was centrifuged at 13000 rpm for 10 min and the supernatant was boiled for 5 min before being loaded onto a 4-20% gradient SDS-PAGE gel. After electrophoresis at 100 V for 75 min, the separated proteins were transferred to an Immobilon-P PVDF membrane (Merck Millipore, Burlington, Massachusetts, USA) at 4 °C and 30 V overnight, and submerged in a blocking buffer (SuperBlock, Thermo Fisher Scientific, Waltham, Massachusetts, USA) for 1 h. The membrane was stained by a primary antibody of choice that targets runt-related transcription factor 1 (Runx2), COL1, osteopontin (OPN), osteocalcin (OCN), β-actin, heat shock protein 47 (HSP47), or Smad 1/2/4 for 1 h, washed five times with 0.1% Tween-20 in TBS, and then incubated with an appropriate HRP-conjugated secondary antibody. Detection was performed by using a Pierce Enhanced Chemiluminescence Western Blotting Substrate Kit (Bio-Rad Laboratories, Hercules, California, USA). The detailed information of the antibodies used in this study is provided as follows: Anti-OPN Rabbit Polyclonal antibody (Sigma-Aldrich, St. Louis, Missouri, USA), Anti-OCN Rabbit Polyclonal antibody (Sigma-Aldrich, St. Louis, Missouri, USA), Anti-Collagen I Rabbit Polyclonal antibody (Abcam, Cambridge, UK), Anti-Runx2 Rabbit Polyclonal antibody (Abcam, Cambridge, UK), Anti-HSP47 Rabbit Monoclonal antibody (Clone: EPR4217; Abcam, Cambridge, UK), Anti-β-Actin Mouse Monoclonal antibody (Clone: mABcam8226; Abcam, Cambridge, UK), Anti-Smad1 Rabbit Monoclonal Antibody (Clone: EP565Y; Abcam, Cambridge, UK), Anti-Smad2 Rabbit Monoclonal Antibody (Clone: EP567Y; Abcam, Cambridge, UK), Anti-Smad1 Rabbit Monoclonal Antibody (Clone: EP618Y; Abcam, Cambridge, UK).

### Cell number determination using Cell Counting Kit-8 (CCK-8)

CCK-8 assay was performed based on a previously described protocol 26, 27. Briefly, infected C3H10T1/2 cells were grown as described above for 5 d, harvested by trypsinization, and then serially diluted in serum-free BME. Then, 100 µL of each dilution was transferred to a clean 96-well microtiter plate, followed the addition of 10 µL of CCK-8 Solution (Dojindo Molecular Technologies, Kumamoto, Japan) to each well. The plate was incubated at 37 °C under a humidified atmosphere containing 5% CO_2_ for 2 h and scanned at 450 nm on a UV-Vis microplate spectrophotometer.

### Hoechst staining

Infected C3H10T1/2 cells were cultivated as described above, harvested, washed with phosphate-buffered saline (PBS), and fixed with a mixed solvent of 75% (v/v) methanol and 25% (v/v) acetic acid for 30 min. The fixed cells were washed with PBS for 5 min and stained with Hoechst 33258 Solution (Solarbio, Beijing, China) at room temperature for 15 min. After staining, the cells were washed with PBS three times for 5 min each, and observed under a fluorescence microscope in a mounting medium of 10% (v/v) glycerol in PBS. The percentage of dividing cells per 100 cells was measured and shown in histograms.

### Cell cycle analysis

Adenovirus-infected C3H10T1/2 cells were cultivated as described above, harvested, washed with PBS, and fixed with 70% ice-cold ethanol at 4 °C for 48 h. The fixed cells were washed with 100 µg/mL Ribonuclease H in PBS, incubated at 37 °C for 30 min, stained with 20 µg/mL propidium iodide in PBS in the dark for 30 min, and subsequently analyzed at 488 nm on a flow cytometer (BD LSR, Franklin Lakes, New Jersey, USA). DNA was quantitated by using Modfit 2.0 software (Verity Software House, Topsham, Maine, USA).

### *In vivo* bone formation

All animal experiment procedures were approved by the Ethics Committee of the Second Affiliated Hospital, Chongqing Medical University (No. 2019-340). Fifteen athymic nude (nu/nu) mice (Harlan Laboratories, Indianapolis, Indiana, USA) aged four to six weeks were randomly divided into three equal-sized experiment groups, including the TGFβ1/BMP9 group, the TGFβ1 group and the BMP9 group. All mice were housed under normal conditions, and were offered food and water ad libitum. C3H10T1/2 cells were infected and then cultivated as described above for 20 h. The infected cells were then collected and resuspended in PBS. The mice were anesthetized with isoflurane and the corneal blink reflex was checked to ensure that all animals were under complete anesthesia. Then, the resuspended cells in PBS were injected into the gastrocnemius muscle on the murine tibia at a dose of 5 × 10^6^ per animal. Specifically, the TGFβ1/BMP9 group were injected with TGFβ1/BMP9-transduced C3H10T1/2 cells, the TGFβ1 group with cells that were infected with AdTGFβ1-RFP and AdGFP, and the BMP9 group with cells that were transduced with AdBMP9-GFP and AdRFP. After five weeks, the mice were euthanized by cervical dislocation and the legs were amputated for microCT imaging. The tissue masses were then resected from the legs and analyzed by hemotoxylin and eosin (H&E) staining.

### Protein microarray analysis

Protein microarray analysis was conducted by Wanyen Biotechnologies (Shanghai, China), using a custom-made Phospho Explorer Antibody Array (Full Moon BioSystems, Sunnyvale, CA, USA). The microarray consisted of 304 antibodies against 144 non-phosphorylated and 157 phosphorylated proteins with six technical replicates each. For sample preparation, adenovirus-infected C3H10T1/2 cells were cultivated as described above and harvested on Day 4. C3H10T1/2 cells were lysed in the Extraction Buffer and loaded onto a separation column pre-equilibrated with Labeling Buffer for 60 min, followed by centrifugation at 750 g for 2 min to collect the protein flow-through. After BCA quantification, 50 μg of the whole cell protein were diluted in Labeling Buffer to a final concentration of 75 μL and incubated with 3 μL of 10 μg/mL Biotin Reagent in DMF for 2 h at room temperature with vigorous vortexing. Then, 35 μL of Stop Reagent were added and the resultant mixture was vortexed for 30 min. Microarray analysis was performed according the manufacturer's instructions. The array slides were visualized on a SureScan Dx Microarray Scanner (Agilent Technologies, Santa Clara, CA, USA). Data were analyzed and normalized by GenePix Pro (version 6.0; Molecular Devices, San Jose, CA, USA). Outliers were removed by employing the Grubb's test. Based on the normalized data, the levels of each protein in the TGFβ1/BMP9 group and the BMP9 group were calculated.

### Kyoto Encyclopedia of Genes and Genomes (KEGG) pathway analysis

KEGG pathway analysis was conducted via the KEGG Automatic Annotation Server (https://www.genome.jp/kegg/kaas). A network of KEGG pathways that were altered between the TGFβ1/BMP9 group and the BMP9 group was constructed with the Pathview Web server (https://pathview.uncc.edu/) based on the microarray data and shown as heat maps.

### Statistical analysis

Statistical analyses were conducted using SPSS 11.0 (IBM, Armonk, New York, USA). Quantitative assays were performed in triplicate and/or repeated in at least three independent experiments. Data were expressed as mean ± SD. Student's t-test was used to evaluate the statistical significant differences between two experimental groups. P < 0.05 was considered statistically significant.

## Results

### Increased expression of TGFβ1 promotes osteogenic differentiation and up-regulates the expression of HSP47 in BMP9-stimulated C3H10T1/2 cells

**Figure 1:**
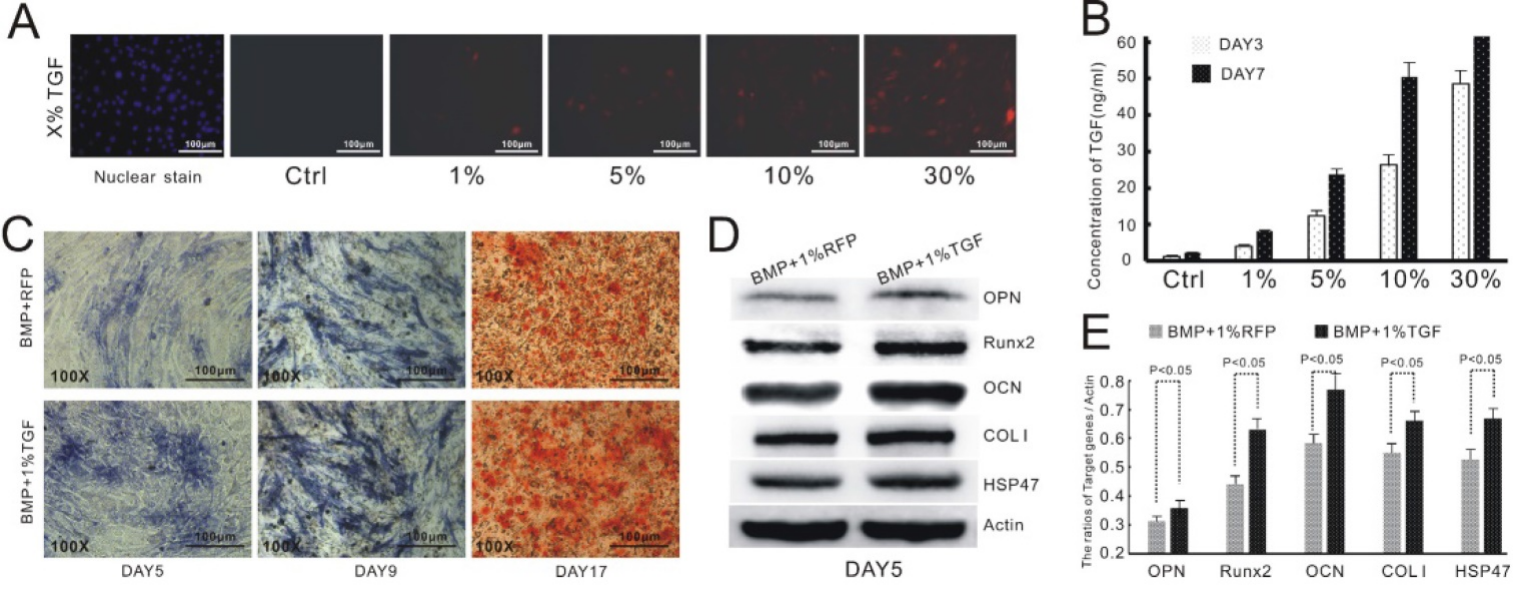
TGFβ1 promotes osteogenic differentiation and HSP47 expression in BMP9-induced C3H10T1/2 cells. **A)** Infection of C3H10T1/2 cells with AdTGFβ1-RFP at various levels of transduction efficiency. **B)** Correlation between the concentration level of TGFβ1 and the efficiency of adenoviral infection. **C)** TGFβ1/BMP9-stimulated C3H10T1/2 cells exhibits increased expression of ALP (day 5 and 9, left and middle) and formation of mineralized calcium nodules (day 17, right) compared to those transduced with BMP9 and RFP. **D)** Western blot images showing increased protein levels of OPN, Runx2, OCN, COL1 and HSP47 in BMP9-induced C3H10T1/2 cells as a result of TGFβ1 overexpression. β-Actin is used as a control. **E)** Densitometric quantitation of the Western blot results.

We have previously reported that TGFβ1 exerts an inhibitory effect on BMP9-mediated osteogenic differentiation of MSCs at concentrations above 20 ng/mL 22. Nevertheless, the study employed the supernatant prepared from a culture of TGFβ1-overexpressing cells, which was ill-suited for *in vivo* validation. To address this drawback, we opted to induce the up-regulation of TGFβ1 in C3H10T1/2 cells via adenoviral transduction. We needed to first establish an appropriate transfection protocol to ensure that the cellular level of TGFβ1 would not exceed 20 ng/mL. To this end, we infected C3H10T1/2 cells with recombinant adenoviruses encoding TGFβ1 and RFP. We then estimated the transfection efficiency based on the fluorescence intensity of the cells (Figure 1A) and measured the concentration of TGFβ1 in the culture supernatant via ELISA at day 3 and 7 after the adenoviral infection (Figure 1B). Base on the experimental data, we concluded that the transfection efficiency needed to be at 1% or less to avoid excessive expression of TGFβ1.

We then verified the effect of TGFβ1 on BMP9-dependent osteogenesis of C3H10T1/2 cells. Consistent with the previous study, the cells infected with both AdTGFβ1-RFP and AdBMP9-GFP (TGFβ1/BMP9 group) showed a considerably higher level of ALP, as evidenced by the purple stain, than those transduced with AdBMP9-GFP and AdRFP (BMP9 group; Figure 1C). Alizarin red staining demonstrated that the formation of mineralized calcium nodules was much more evident in the TGFβ1/BMP9 group (Figure 1C). Quantitative analysis of osteogenic markers by Western Blotting indicated that TGFβ1 significantly up-regulated the protein levels of OPN, Runx2, OCN and COL1 in BMP9-transduced cells. Importantly, the TGFβ1/BMP9-stimulated cells also exhibited significantly augmented expression of HSP47. Taken together, the experimental data confirmed the synergy between TGFβ1 and BMP9 in promoting the osteogenic differentiation of C3H10T1/2 cells.

### TGFβ1 suppresses the proliferation of BMP9-induced C3H10T1/2 cells

**Figure 2:**
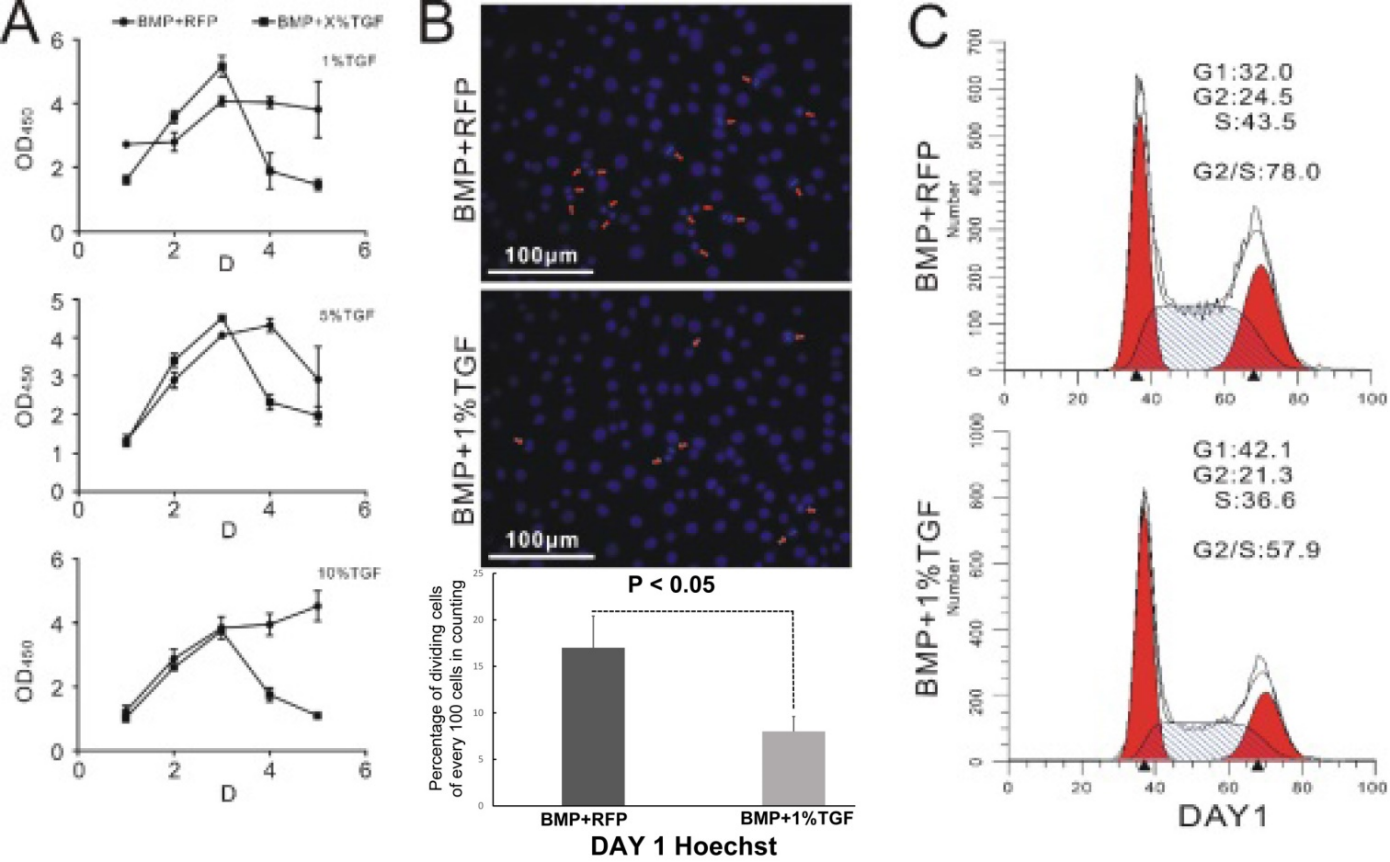
TGFβ1 inhibits the proliferation of BMP9-induced C3H10T1/2 cells. **A)** Monitoring of the growth curve of BMP9/RFP- (circle) or TGFβ1/BMP9- (square) transduced C3H10T1/2 cells by CCK-8 analysis. **B)** Hoechst staining (showing the percentage of dividing cells) and **C)** flow cytometry-based cell cycle analysis of BMP9/RFP- (top) or TGFβ1/BMP9- (bottom) transduced C3H10T1/2 cells.

It is well known that stem cells maintain a delicate balance between self-renewal and differentiation 28, 29. Therefore, we hypothesized that TGFβ1 should exert an inhibitory effect on the proliferation of BMP9-induced C3H10T1/2 cells. This was indeed supported by the measurement of cell count via CCK-8 assay. As illustrated in Figure 2A, cells in the TGFβ1/BMP9 group proliferated at a similar rate as those in the BMP9 group during the first three days after the adenoviral infection. However, starting from day 3, the number of proliferative cells in the TGFβ1/BMP9 group declined rapidly, whereas that of the BMP9 group either showed a moderate drop or remained largely stable. As a result, at day 4 and day 5, there were significantly fewer proliferative cells in the TGFβ1/BMP9 group compared to the BMP9 group, regardless of the transduction efficiency of TGFβ1 (1%, 5% or 10%). The difference in the level of cell proliferation between the two groups was further corroborated by Hoechst staining, which showed fewer TGFβ1/BMP9-transduced C3H10T1/2 cells undergoing mitosis than those induced with only BMP9 on day 1 (Figure 2B). Moreover, cell cycle analysis based on propidium iodide staining revealed that stimulation with TGFβ1 significantly reduced the percentage of BMP9-induced C3H10T1/2 cells in the G2/S phase (Figure 2C). Combined, these experimental results demonstrated that TGFβ1 could inhibit the proliferation of BMP9-overexpressing C3H10T1/2 cells.

### TGFβ1 enhances BMP9-dependent bone formation from C3H10T1/2 cells in mice

**Figure 3:**
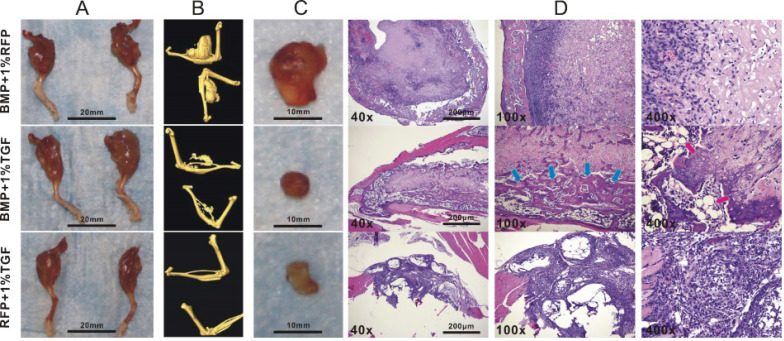
TGFβ1 enhances bone formation from BMP9-stimulated C3H10T1/2 cells in mice. Athymic nude (nu/nu) mice aged four to six weeks were injected intramuscularly into the gastrocnemius muscle on their tibiae with BMP9/RFP-, TGFβ1/GFP-, or TGFβ1/BMP9-transduced C3H10T1/2 cells. **A)** Amputated murine legs bearing ectopic bone matrices and **B)** microCT imaging results. **C)** Resected ectopic bone matrices. **D)** H&E staining showing the presence of periosteum, adipocytes (red arrows) and thicker trabecular structures (blue arrows) in the osteroid sections prepared from the TGFβ1/BMP9 group.

Although TGFβ1 has been shown to promote the osteogenic differentiation of BMP9-induced C3H10T1/2 cells, the lack of animal studies makes it difficult to determine whether such effects could lead to enhanced bone formation *in vivo*. We thus investigated whether TGFβ1-dependent osteo-induction that we observed on a cellular level could be replicated in a mouse model. To this end, we intramuscularly injected different types of adenovirally transduced C3H10T1/2 cells into the gastrocnemius muscle on the murine tibiae and examined the resultant development of ectopic masses on the legs. MicroCT imaging indicated that the average size of the ectopic masses for the BMP9 group was significantly greater than that for the TGFβ1/BMP9 group or the TGFβ1 group (Figure 3A, B). This was in agreement with the anti-proliferative effect of TGFβ1 described earlier. The ectopic bone matrices were then carefully separated from the masses of legs and examined further. It was found that, unlike the ectopic bone matrices in the other two groups, those resected from the TGFβ1 group were composed primarily of fibrous rather than bone tissues (Figure 3C), echoing earlier observations that TGFβ1 alone could not induce osteogenesis in pluripotent mesenchymal cells 30. H&E staining further confirmed that tissue sections prepared from the TGFβ1 group consisted mainly of fibroblasts and lacked any meaningful bone structures, whereas the bone matrices from the BMP9 group contained intact periosteum and visible trabeculae (Figure 3D). In comparison, the ectopic bone matrices from the TGFβ1/BMP9 group developed more mature osteoid structures, characterized by the presence of thicker trabeculae, periosteum and adipocytes (Figure 3D). Combined, these results provided further evidence that TGFβ1 could facilitate the formation and maturation of bone tissues from BMP9-induced C3H10T1/2 cells.

### HSP47 could mediate the osteogenic effect of TGFβ1 on BMP9-stimulated C3H10T1/2 cells

**Figure 4:**
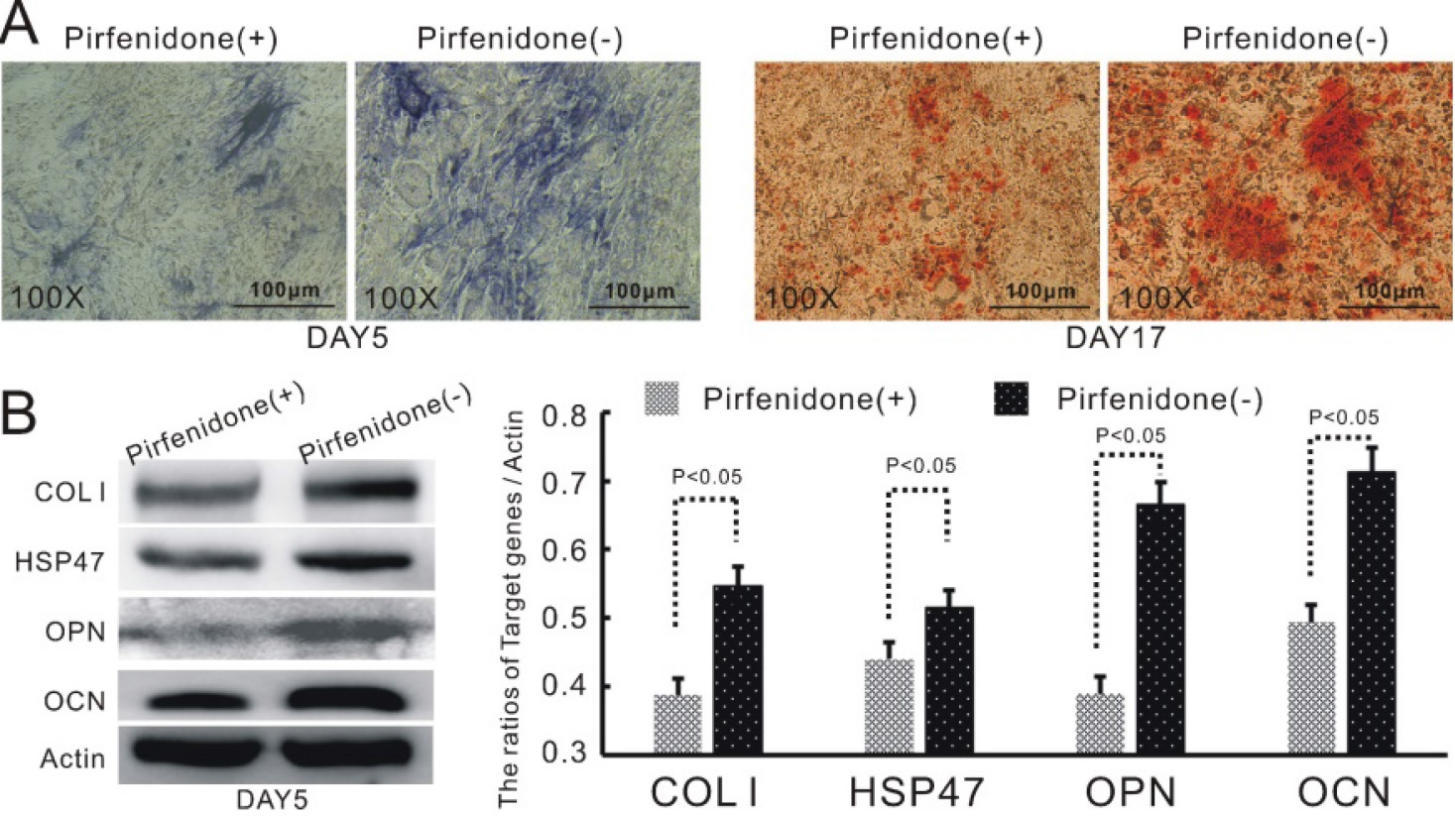
HSP47 mediates TGFβ1-dependent up-regulation of osteogenic differentiation in BMP9-induced C3H10T1/2 cells. Inhibition of HSP47 by pirfenidone significantly attenuates the stimulatory effects of TGFβ1 on **A)** the level of ALP (left) and the formation of mineralized calcium nodules (right), as well as **B)** the protein expression of COL1, HSP47, OPN and OCN in BMP9-induced C3H10T1/2 cells (left) and densitometric quantitation of the Western blot results (right).

As described earlier, we have discovered that the up-regulation of TGFβ1 in BMP9-induced C3H10T1/2 cells led to increased expression of COL1 and its up-stream modulator HSP47. HSP47 has been previously reported to play a key role in the folding and molecular maturation of COL1. Thus, we speculated that HSP47 might mediate TGFβ1-dependent osteogenic differentiation of BMP9-induced C3H10T1/2 cells. Indeed, treatment of TGFβ1/BMP9-transduced C3H10T1/2 cells with pirfenidone, a well-established inhibitor of COL1 and HSP47 31, 32, was found to markedly attenuate the production of ALP and the formation of mineralized calcium nodules (Figure 4A). Moreover, pirfenidone could also significantly diminish the protein levels of OPN and OCN (Figure 4B). Taken together, these data provided evidence that increased expression of HSP47 could be a contributing factor to the enhanced osteogenic differentiation of TGFβ1/BMP9-stimulated C3H10T1/2 cells that we observed in this study.

### Treatment of BMP9-induced C3H10T1/2 cells with TGFβ1 results in activation of non-canonical signaling pathways

**Figure 5:**
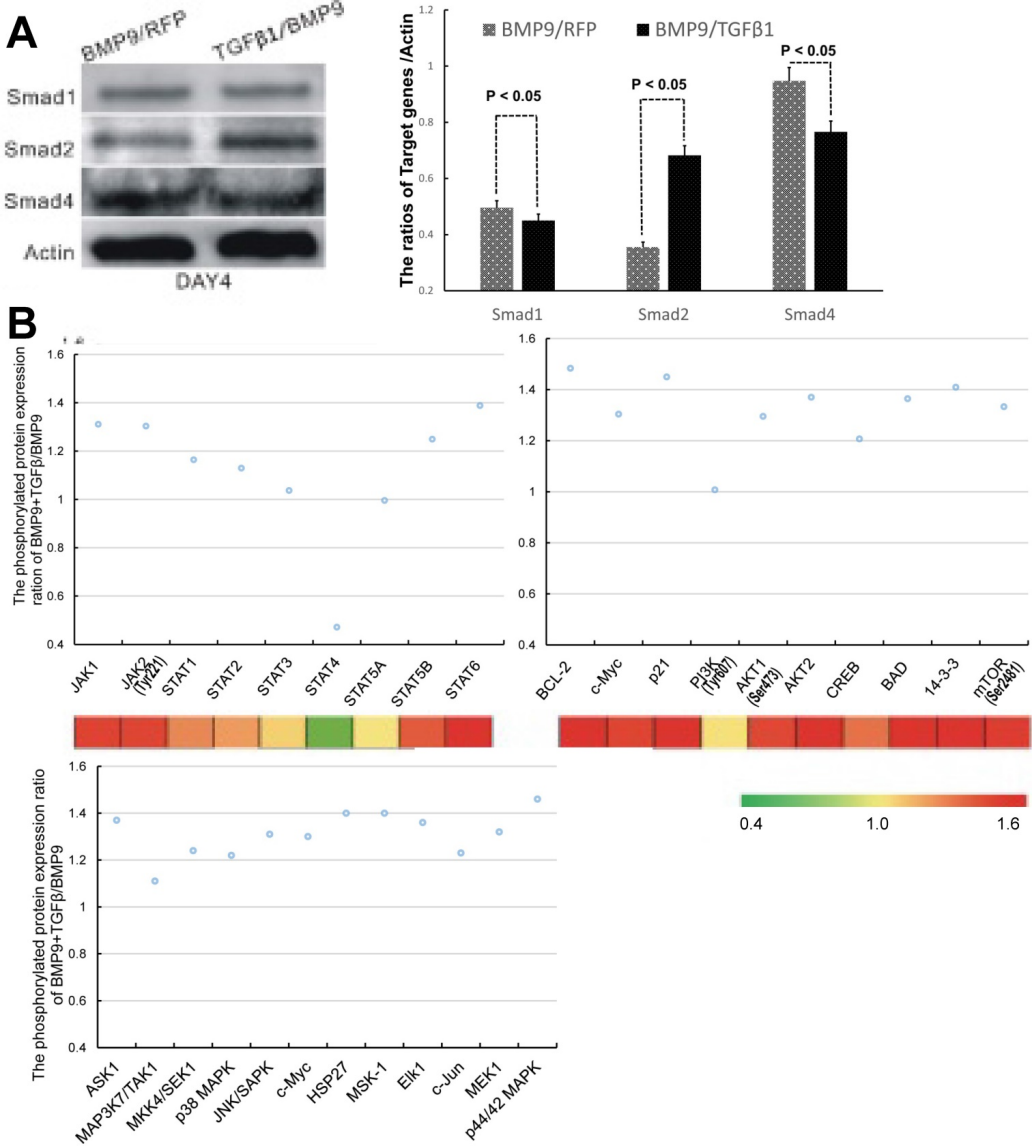
TGFβ1 might exert its osteogenic effects in BMP9-induced C3H10T1/2 cells via non-canonical signaling pathways. **A)** Comparison of the protein levels of Smad1, Smad2 and Smad4 between BMP9/RFP- (left, gray) and TGFβ1/BMP9- (right, black) transduced C3H10T1/2 cells. **B)** The phosphorylation levels of selected proteins involved in the JAK-STAT, PI3K-AKT and MAPK signaling pathways.

We next sought to probe the molecular mechanisms responsible for the regulatory effect of TGFβ1 on the osteogenic differentiation of BMP9-stimulated C3H10T1/2 cells. Since TGFβ/Smad was the classical osteogenic signaling pathway, we first examined the expression of Smad1, Smad2 and Smad4, which are the downstream targets of BMP9 33, TGFβ1 19 and TGFβ1/BMP9 34, respectively. The experimental data that we obtained indicated that the TGFβ1/BMP9-tranduced C3H10T1/2 cells had a statistically comparable level of Smad1 and significantly greater level of Smad2 compared to those transduced with BMP9 only (Figure 5A). It came as a surprise that the TGFβ1/BMP9 group showed a significantly reduced level of Smad4 than the BMP9 group. Since the BMP9 and TGFβ1 signaling pathways share heterodimeric complexes of type I and II dual-specificity kinase receptors 35, it is possible that increased level of TGFβ1 might suppress BMP9 regulation of Smad4 through competitive inhibition. At the same time, the lower protein level of Smad4 in the TGFβ1/BMP9 group implied that TGFβ1 could have activated the osteogenic differentiation of C3H10T1/2 cells by non-canonical signaling pathways 36, 37.

To probe whether non-canonical signaling mechanisms were involved, we performed microarray analysis to compare between the TGFβ1/BMP9 group and the BMP9 group the expression levels of 304 proteins, including 157 phosphorylated proteins, that are implicated in 16 common pathways associated with cell cycle, adhesion, proliferation, differentiation, apoptosis, etc. Among the phosphorylated proteins that we analyzed, 72 were significantly up-regulated in the TGFβ1/BMP9-transduced cells. KEGG pathway analysis further revealed that a significant number of proteins in the JAK-STAT, PI3K-Akt, and MAPK signaling pathways showed enhanced phosphorylation in the TGFβ1/BMP9 group compared to the BMP9 group (Figure 5B). Therefore, these signaling pathways might play important mechanistic roles in TGFβ1/BMP9-stimulated bone formation from C3H10T1/2 cells.

## Discussion

Our current study demonstrated that adenovirally induced up-regulation of TGFβ1 simultaneously promoted the osteogenic differentiation and attenuated the proliferation of BMP9-induced C3H10T1/2 BMSCs. Importantly, the use of adenoviral vectors allowed us to generate cells that stably overexpressed TGFβ1 for *in vivo* investigations. Transplantation of the TGFβ1/BMP9-activated C3H10T1/2 cells into the gastrocnemius muscle of murine tibia led to significant ectopic bone matrices with relatively mature osteoid structures. In comparison, those derived from the BMP9-transduced cells contained less periosteum and trabeculae. These results provided further insight into the synergy between TGFβ1 and BMP9 in promoting MSC osteogenesis.

Surprisingly, the TGFβ1-BMP9 synergy does not seem to act through increased expression of Smad4. Wu et al. have reported that BMPR2 and ActR2, two type II TGFβ1 receptors, might be implicated in mediating BMP9-dependent osteogenesis of C3H10T1/2 cells 38. Previous investigations have revealed that BMP2 could facilitate the heterodimerization of BMPR2 and BMPR1A by binding to both subunits, which in turn could elevate the expression of Smad4 36. Because BMP9 has also been shown to interact with BMPR2 in modulating the formation of pulmonary vasculature 39-41, it is possible that a similar regulatory mechanism might be involved in the activation of Smad4. In fact, these findings are consistent with our observation that C3H10T1/2 cells infected with BMP9 exhibited greater expression of Smad4 compared to those transduced with both TGFβ1 and BMP9. Since TGFβ1 is known to interact with BMPR2, the apparent attenuation of Smad4 expression by TGFβ1 in BMP9-induced BMSCs could be the result of competitive inhibition.

Because our initial data failed to produce definitive evidence that TGFβ1-dependent osteogenesis of BMP9-induced C3H10T1/2 cells was mediated by Smads, we conducted a protein microarray study to probe whether other signaling pathways were involved. Our results suggested that the JAK-STAT, PI3K-Akt and p38 MAPK signaling pathways were activated in the TGFβ1/BMP9 group compared to the BMP9 group. JAK-STAT is primarily known to modulate cytokine signaling 42 but has recently been shown to play a potential role in bone metabolism 43. There is evidence that the JAK2-STAT5B signaling axis could potentially be implicated in osteoblastic differentiation via its modulation of downstream effectors such as Runx2 44, 45. Dieudonne et al. demonstrated that interaction of casitas B-linage lymphoma with STAT5 and the subsequent association with Runx2 could augment MSC osteogenesis 46. On the other hand, experimental findings by Genetos and coworkers indicated that proliferation and osteogenic differentiation of pre-osteoblasts were markedly up-regulated by annexin A2 or A5-dependent activation of STAT6 47. These findings are consistent with our observation of significantly increased expression levels of STAT6 and STAT5B as a result of TGFβ1 activation.

It is well established that the activation of the PI3K-Akt axis by TGFβ1 can trigger a wide range of metabolic and cellular effects in different cell types 48-51. Consistent with our finding, the PI3K-Akt cascade has long been considered as one of the most significant Smad-independent regulatory route for MSC osteogenesis 52. For example, Zhang et al. has reported that treatment with 1 ng/mL of TGFβ1 augmented the proliferation and mineralization of human hFOB1.19 osteoblasts via the activation of PI3K-Akt 53. On the other hand, there is also evidence of BMP9-induced enhancement of the PI3K-Akt signaling pathway in an osteo-stimulatory context. In Furue et al.'s study, inhibition of PI3K was found to be able to significantly attenuate BMP9-dependent up-regulation of ALP and other key osteogenic markers 54. Furthermore, Eiraku and colleagues showed that BMP9 could accentuate the phosphorylation of Akt and glycogen synthase kinase 3-β independently of Wnt, resulting in increased expression of ALP and Runx2. It is also interesting to note that PI3K-Akt might also mediate the activation of β-catenin by TGFβ1 in human MSCs 55. Inhibition of β-catenin has been demonstrated to attenuate BMP9-induced up-regulation of osteogenic markers in MSCs 56, whereas another study indicated that knockdown of β-catenin could mitigate the inhibitory effect of TGFβ1 on the production of bone sialoprotein 57. As a result, β-catenin seems to demonstrate a complex mechanistic relationship with TGFβl that echoes the latter's own biphasic regulation of BMP9-stimulated MSCs. Combined, these findings implied that the interplay between TGFβl/BMP9 and PI3K-Akt could be at least partially responsible for stimulating the osteogenic differentiation of MSCs.

HSP47 is a collagen-specific glycoprotein that belongs to the serpin family, and has been found to play a critical role in collagen maturation 58, 59. Importantly, HSP47 has been regularly shown to be targeted by TGFβ1 in fibrosis. For example, TGFβ1 has been reported to elevate both the mRNA and protein levels of HSP47 and COL1 in human lung fibroblasts, and such effects can be attenuated by pirfenidone 31. TGFβ1-induced stimulation of HSP47 was also observed in the stromal fibroblasts obtained from the conjunctival tissues of patients with ocular cicatricial pemphigoid 60. A survey of past studies implied that the activation of HSP47 by TGFβ1 might involve a set of complex and even opposing mechanisms. Xiao and colleagues revealed that suppression of JNK could block TGFβ1-induced activation of HSP47 and the resultant increase of extracellular matrix (ECM) synthesis in a human proximal tubular epithelial cell line HK-2 61. In another study, Stambe and coworkers revealed that the stimulatory effect of TGFβ1 on collagen synthesis could be attenuated by the inhibition of p38, which concomitantly diminished the protein level of HSP47 62. These results coincided with the increased phosphorylation levels of p38 and JNK that we observed in the TGFβ1/BMP9 group compared to the BMP9 group. On the other hand, it should also be pointed out that the canonical Smad signaling pathway might also play an important role, as evidenced by Kim et al.'s finding that Smad2/3 was mechanistically implicated in TGFβ1-dependent up-regulation of HSP47 and ECM production in nasal fibroblasts 63. As elucidated earlier, enhanced production of COL1 could lead to the development of a well-connected collagen fiber network, which is beneficial for bone formation, by facilitating calcium deposition and osteoid maturation. Therefore, we speculated that HSP47-mediated augmentation of collagen synthesis by TGFβ1 could synergize with BMP9-dependent bone apposition and mineralization.

In conclusion, our study demonstrated that TGFβ1 could promote the osteogenic differentiation and inhibit the proliferation of BMP9-induced C13H10T1/2 cells, which, when replicated in mice, resulted in enhanced bone formation characterized by a more mature collagen-matrix structure and a greater extent of calcium mineralization. We showed that HSP47, which modulates COL1 expression, was up-regulated by TGFβ1, whereas inhibition of HSP47 by pirfenidone significantly mitigated the osteogenic effects of TGFβ1. Furthermore, our microarray studies suggested that the observed enhancement of bone formation might act through non-canonical signaling pathways. These results provided useful insights into the molecular mechanisms responsible for TGFβ1-dependent osteo-induction of BMSCs.
